# Supplementary material for: Human Embryonic-Derived Mesenchymal Progenitor Cells (hES-MP Cells) are Fully Supported in Culture with Human Platelet Lysates
Source: Bioengineering (Basel). 2020 Jul 20;7(3):75. doi: 10.3390/bioengineering7030075 (PMC7552691; doi:10.3390/bioengineering7030075)
Supplement: Supplementary file 1 [file bioengineering-07-00075-s001.pdf]

Supplementary Materials

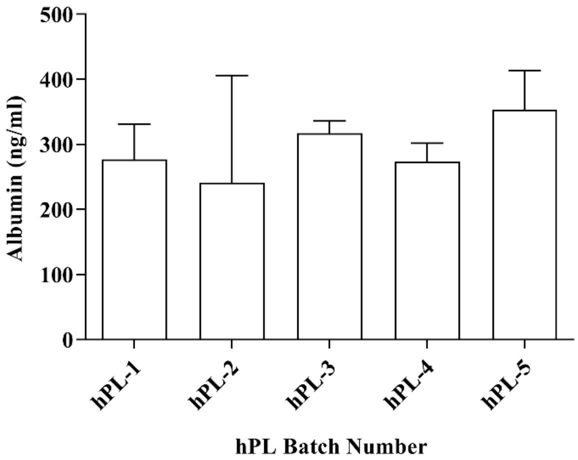

Figure S1. Human serum albumin concentrations for different hPL batches. No significant batch variability was detected between five different hPL batches.

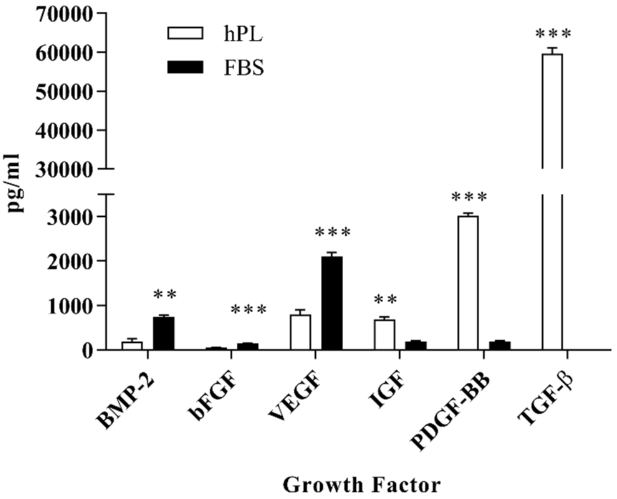

Figure S2. Concentrations of key growth factors in hPL (n = 5) and FBS (n = 3) treatments. Concentrations of BMP-2, bFGF, and VEGF were significantly higher in FBS than in hPL. Concentrations of IGF, PDGF-BB, and TGF-β were significantly higher in hPL than in FBS**.** (** $p \leq 0.01$; *** $p \leq 0.001$)

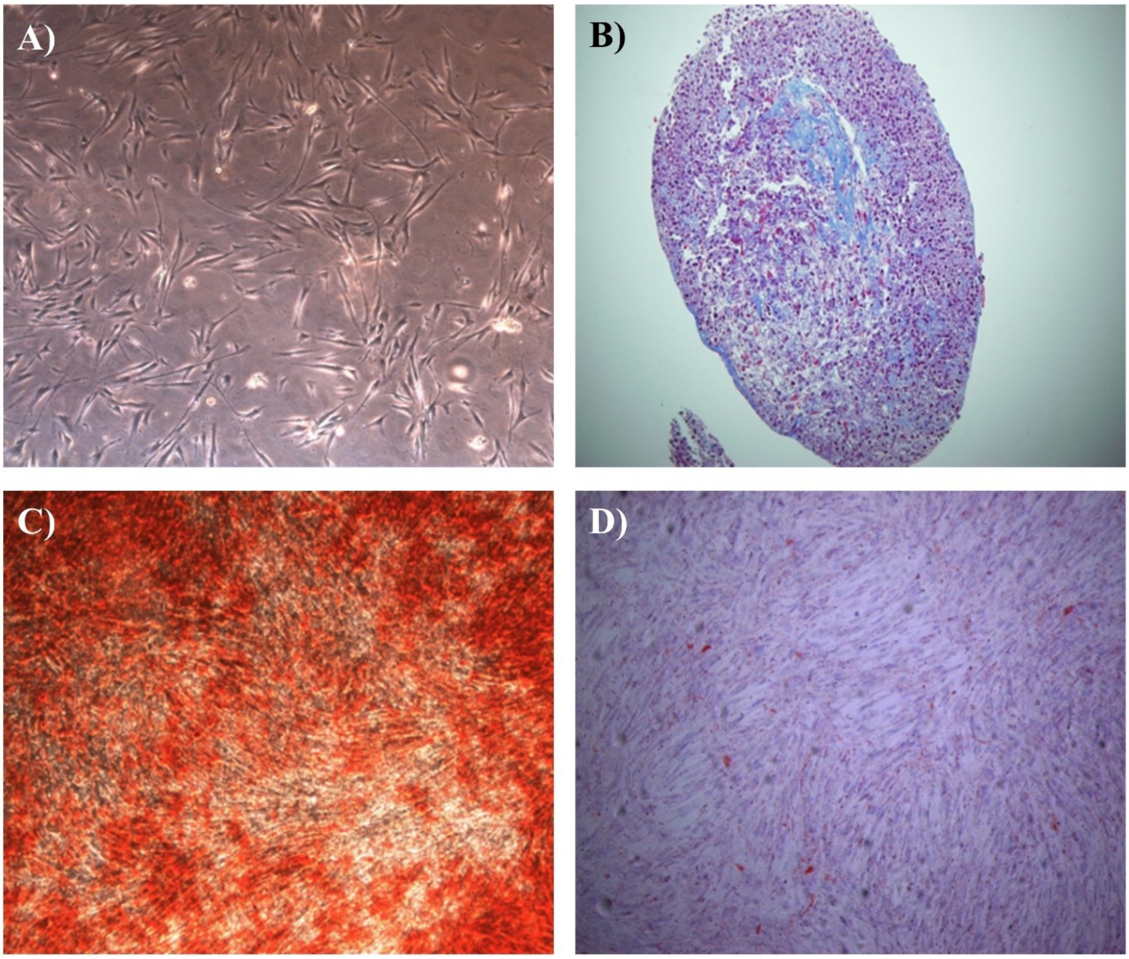

Figure S3. Morphology and trilineage differentiation of hES-MP cells. A) Morphology after 7 days of expansion. B) Chondrogenic differentiation after 35 days; Masson Trichrome blue staining. C) Osteogenic differentiation after 28 days; alizarin red staining. D) Adipose differentiation after 14 days; oil red O staining.

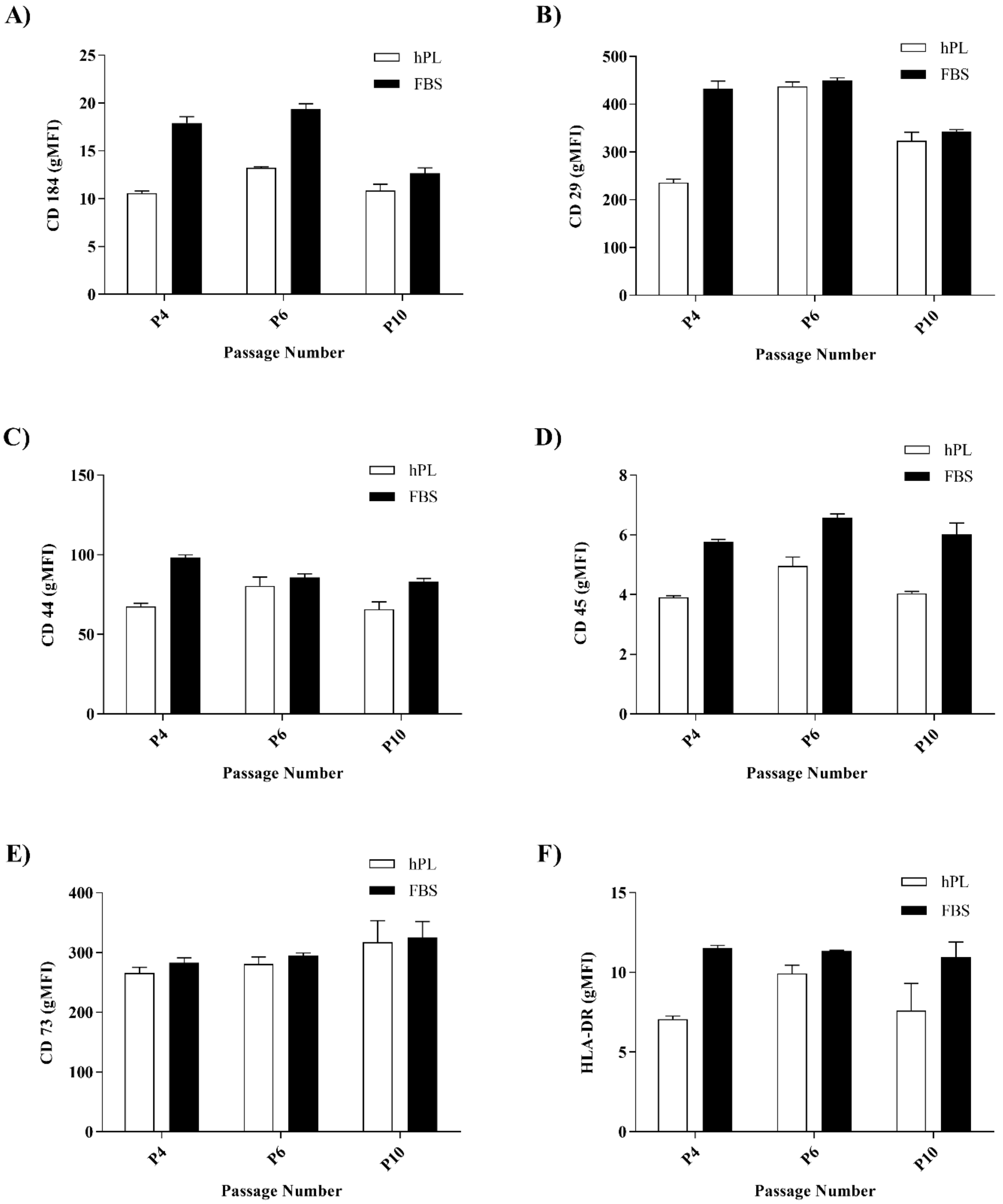

Figure S4. Surface marker expression on hES-MP cells following culture in hPL. hES-MP cells expressed CD29, CD44, and CD73 (B, C, and E). Expression of CD45, CD184, and HLA-DR (A, D, and F) was at very low levels only (e.g. under 20 gMFI). No significant differences in expression were found related to the type of culture supplement (hPL or FBS) used. (gFMI = geometric mean fluorescence intensity)

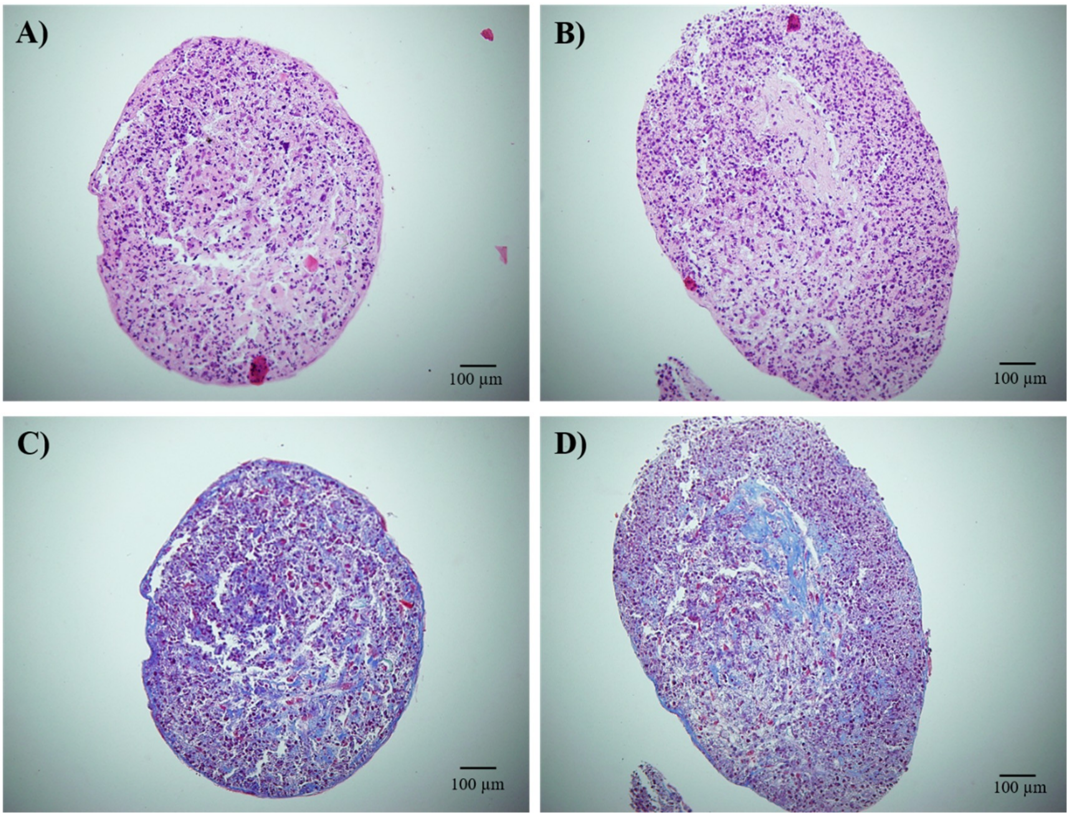

Figure S5. hES-MP cartilage pellets after 28 days of differentiation. hES-MP cells were grown in either FBS (A and C) or hPL (B and D). Cartilage pellets were stained with haematoxylin and eosin stain (A and B) and Masson's trichrome stain (C and D). For both FBS- (A and C) and hPL-derived (B and D) hES-MP pellets, hypertrophic cells could be observed within the pellets, as could deposition of extracellular matrix that stained blue with Masson's trichrome stain (C and D).
